# A novel compound heterozygous NBAS variant with HLH and multisystem involvement: expanding the clinical spectrum and literature review

**DOI:** 10.3389/fimmu.2026.1829362

**Published:** 2026-06-01

**Authors:** Xiaozhen Gong, Ye Feng, Qianlu Zhang, Lin Tong, Zijuan Feng, Xiaodong Zhao, Lina Zhou, Ying Dou

**Affiliations:** 1 National Clinical Research Center for Children and Adolescents' Health and Diseases, Children’s Hospital of Chongqing Medical University, Chongqing, China; 2 Ministry of Education Key Laboratory of Child Development and Disorders, Children’s Hospital of Chongqing Medical University, Chongqing, China; 3 Chongqing Key Laboratory of Child Rare Diseases in Infection and Immunity, Children’s Hospital of Chongqing Medical University, Chongqing, China; 4 Department of Hematology & Oncology, Children’s Hospital of Chongqing Medical University, Chongqing, China; 5 Department of Rheumatology & Immunology, Children’s Hospital of Chongqing Medical University, Chongqing, China

**Keywords:** hematologic abnormalities, HLH, ILFS-2, immunodeficiency, NBAS, short stature

## Abstract

Biallelic variants in the neuroblastoma amplified sequence (NBAS) gene have been associated with short stature, optic atrophy, and Pelger-Huët anomaly (SOPH) syndrome, infantile liver failure syndrome type 2 (ILFS-2), and an increasingly broad spectrum of clinical phenotypes. However, cases presenting with hemophagocytic lymphohistiocytosis (HLH) accompanied by immune dysfunction and multisystem involvement have not been reported. We identified novel compound heterozygous variants in the NBAS gene, c.5139-5T>G and c.5983C>T, in a Chinese girl who successively developed recurrent infections, short stature, ILFS-2, progressive cytopenias from leukopenia to bicytopenia and eventually pancytopenia, and HLH. Polymerase chain reaction confirmed that c.5139-5T>G variant caused aberrant splicing. The c.5983C>T variant is novel and was classified as likely pathogenic according to American College of Medical Genetics and Genomics guidelines. Western blotting of the patient’s PBMCs detected only a truncated NBAS protein, consistent with the product predicted from the c.5983C>T variant. Functional studies in HEK293T cells overexpressing the truncated NBAS protein further indicated impaired NBAS function, as assessed by real-time quantitative reverse transcription polymerase chain reaction and immunofluorescence analysis. We systematically reviewed 322 previously reported patients and analyzed genotype–phenotype correlations in 316 cases to further define the clinical spectrum of NBAS-related disease. We identified a novel pathogenic NBAS variant, further expanded the phenotypic spectrum of NBAS-related disease, and provided additional insight into genotype–phenotype correlations. Pancytopenia and HLH may reflect severe immune dysregulation and poor prognosis, highlighting the importance of early recognition and timely intervention.

## Introduction

The neuroblastoma amplified sequence (NBAS) protein is involved in multiple cellular processes, most notably nonsense-mediated decay (NMD) and Golgi-to-endoplasmic reticulum (ER) retrograde transport (1–3). Biallelic variants in NBAS have been implicated in a rare autosomal recessive disorder with marked clinical heterogeneity. NBAS was initially identified as the disease-causing gene in a syndromic condition characterized by short stature, optic nerve atrophy, and Pelger–Huët anomaly (SOPH syndrome), first described in individuals from the Yakut population (4). Subsequent studies have considerably broadened the phenotypic spectrum, and accumulating evidence from published cohorts has shown that biallelic NBAS variants can lead to a wide range of hepatic, skeletal, ocular, and immunologic manifestations (5–11).

Among these manifestations, recurrent episodes of fever-triggered acute liver dysfunction represent one of the most characteristic features of NBAS-associated disease, often beginning in early childhood and corresponding to infantile liver failure syndrome type 2 (ILFS-2) (5). In addition, syndromic features accompanied by variable immune abnormalities have been increasingly recognized (11–17). Hematologic involvement has mainly been reported in the setting of immune dysfunction, most commonly as neutropenia or other mild cytopenias (9, 13, 18–20). By contrast, persistent trilineage cytopenia or profound pancytopenia has not been widely recognized as a core feature of NBAS deficiency.

Hemophagocytic lymphohistiocytosis (HLH) is a life-threatening hyperinflammatory syndrome characterized by uncontrolled immune activation and multiorgan involvement (21). Although HLH is not classically regarded as part of the defining phenotype of NBAS-associated disease, recent genetic studies have identified biallelic NBAS variants in a small number of pediatric HLH cases (22), and several isolated case reports have described HLH in patients with confirmed NBAS deficiency (23, 24). These observations suggest that, although uncommon, HLH may represent a severe inflammatory complication in susceptible individuals. Importantly, the development of HLH is often associated with rapid clinical deterioration, progressive cytopenias, hepatic injury, and a high risk of mortality, underscoring its major prognostic significance.

Here, we report a patient harboring compound heterozygous NBAS variants, including a previously reported splice-site variant and a novel truncating variant. The patient developed a severe multisystem phenotype characterized by short stature, immunodeficiency, liver failure, hematologic abnormalities, and ultimately fatal HLH. Through comprehensive genetic and functional analyzes, we sought to clarify the molecular consequences of these variants and to further refine the clinical understanding of severe inflammatory complications in NBAS-associated disease.

## Materials and methods

### Patient and samples

The blood samples were obtained from residual specimens used for routine clinical testing, and clinical data were collected from routine diagnostic and therapeutic activities. A written informed consent was obtained from the child’s parents. The study was conducted in compliance with the principles of the Declaration of Helsinki and was approved by the Ethics Committee of the Children’s Hospital of Chongqing Medical University on April 13, 2021 (Approval No. [2021] Ethics Review (Research) No. 138).

### Data acquisition, and definition of variables

Published case data were collected using the search terms “NBAS AND/OR SOPH”, “NBAS AND/OR ILFS2” and “NBAS deficiency” on PubMed and Google Scholar from August 2010 to October 2025 (4–15, 18, 19, 22–74). Clinical features were named according to Human Phenotype Ontology [HPO] terminology.

### Genetic analysis

Genomic DNA samples from peripheral blood were extracted by standard procedures using QIAamp DNA Mini Kit (Qiagen, Germany) and submitted to MyGenostics (Beijing, China) for whole-exome sequencing (WES). The identified variants were subsequently confirmed by Sanger sequencing (Shanghai, China).

### Pathogenicity of the NBAS variant

Variant classification followed the American College of Medical Genetics and Genomics (ACMG) guidelines. Allele frequencies for the *NBAS* (NM_015909.4) c.5983C>T (p.Arg1995Ter) variant was retrieved form public population databases, including the 1000 Genomes Project, gnomAD, and ExAC. The in-silico pathogenicity of *NBAS* c.5983C>T variant was assessed via multiple prediction tools (LRT, Mutation Taster, CADD, DANN, FATHMM_MKL, Eigen, and fitCons). Evolutionary conservation of NBAS p.Arg1995 residue (ref.seq NP_056993.2) was assessed using NCBI reference data and multiple-sequence alignment generated with Clustal Omega.

### Western blot analysis

Peripheral blood mononuclear cells (PBMCs) from the patient and two unrelated healthy controls were lysed in RIPA buffer supplemented with PMSF and PIC. Protein extracts were separated by SDS-PAGE, transferred to PVDF membranes (Millipore, Germany), and incubated with primary antibodies against NBAS (Sigma-Aldrich, USA) and GM130 (Proteintech, China), followed by HRP-conjugated secondary antibodies. Signals were detected using enhanced chemiluminescence (Amersham, Sweden).

### Polymerase chain reaction

Total RNA was extracted from the proband’s mother and reverse-transcribed into cDNA according to the manufacturers’ protocols. Primers spanning NBAS cDNA exons 40–45 were as follows: forward, 5′-GCAGTCCCCCTCTGCATTAT-3′; reverse, 5′-AGCACTGCCACCACTCAAT-3′. PCR amplification was performed using Q5 Hot Start High-Fidelity DNA Polymerase (New England Biolabs, USA), and the PCR products were subjected to Sanger sequencing.

### Generation of NBAS mutant expression plasmids

Full-length wild-type (WT) NBAS cDNA was purchased from Youbao Biotechnology (Changsha, China). The c.5983C>T mutant (MUT) NBAS construct was generated by PCR-based site-directed mutagenesis and subcloned into the 7.1-pCMV-3×Flag and pcDNA3.1-eGFP expression vector.

### Real-time quantitative reverse transcription PCR

HEK293T cells were transfected with WT or MUT eGFP-tagged NBAS plasmids using Lipofectamine 3000 (Thermo Fisher Scientific). Total RNA was extracted 24 h after transfection and reverse-transcribed to generate cDNA. RT-qPCR was performed using primers targeting eGFP or NBAS (eGFP: forward, 5′-GACGTAAACGGCCACAAGTTC-3′, reverse, 5′-AAGTCGTGCTGCTTCATGTG-3′; NBAS: forward, 5′-TTTGGCTGCCAGCAGAGAGTAC-3′, reverse, 5′-CTAGCTCCTCTTGAATGGCAGG-3′), with 18S rRNA as the endogenous control. Relative expression levels were calculated using the ΔΔCt method.

### Immunofluorescence analysis

HEK293T cells were transfected with WT or MUT Flag-tagged NBAS plasmids. At 48 h after transfection, cells were seeded onto gelatin-coated coverslips, fixed with 4% paraformaldehyde, permeabilized with saponin (GSBIO, China), and blocked with 2% BSA in saponin. Cells were subsequently incubated with primary antibodies against FLAG (Selleck, China), GM130 (Proteintech, China), and calreticulin (Proteintech, China), followed by appropriate fluorescent secondary antibodies. Confocal images were acquired using a Nikon C2 microscope (Nikon, Japan), and colocalization of NBAS with Golgi and ER markers was quantified using Manders’ overlap coefficients.

### Statistical analysis

Statistical analyzes were performed using GraphPad Prism version 10.1.2 (GraphPad Software, USA). A Student t test was used for comparisons. Data are presented as mean ± SD, ** P < 0.01, *** P < 0.001, **** P < 0.0001.

## Results

### Case presentation

**Figure 1:**
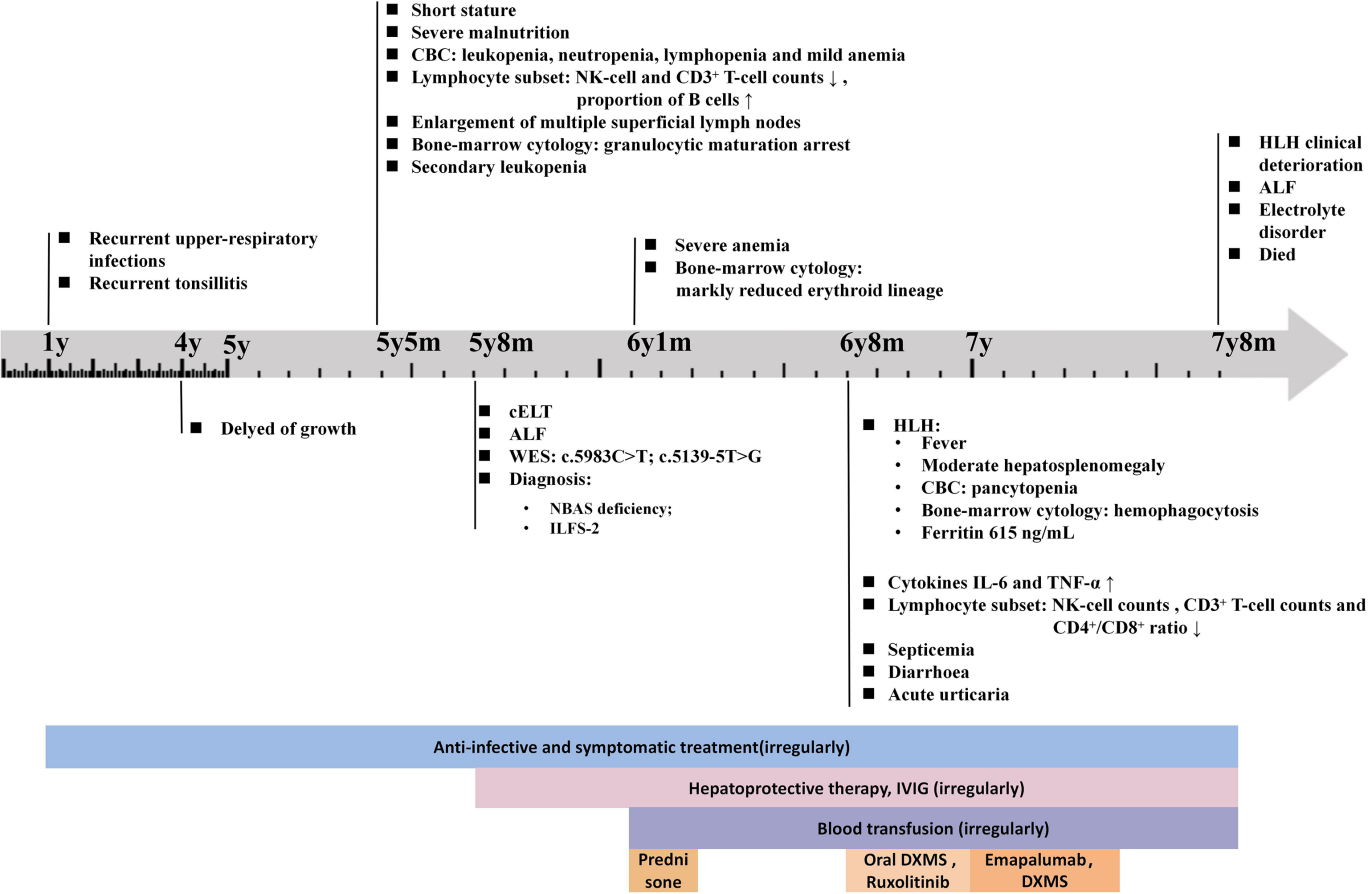
Timeline of the clinical course (including diagnostic workup, therapies, and outcomes). CBC, complete blood count; cELT, continuously elevated liver transaminases; ALF, acute liver failure; WES, whole exome sequencing; HLH, hemophagocytic lymphohistiocytosis.

The proband was born at term to healthy, non-consanguineous Chinese parents, with a birth weight of 2.2 kg. The perinatal and birth history was unremarkable. The timeline of the clinical course is shown in **Figure 1**. From approximately 1 year of age, she experienced recurrent upper respiratory tract infections and tonsillitis, and growth faltering became apparent from around 4 years of age. At 5 years and 5 months, she was first admitted to our hospital because of fever and leukopenia. Complete blood count (CBC) showed leukopenia, neutropenia, and lymphopenia. After inpatient anti-infective treatment, the absolute neutrophil count increased but remained below the normal range, and she was diagnosed with secondary leukopenia.

**Figure 2:**
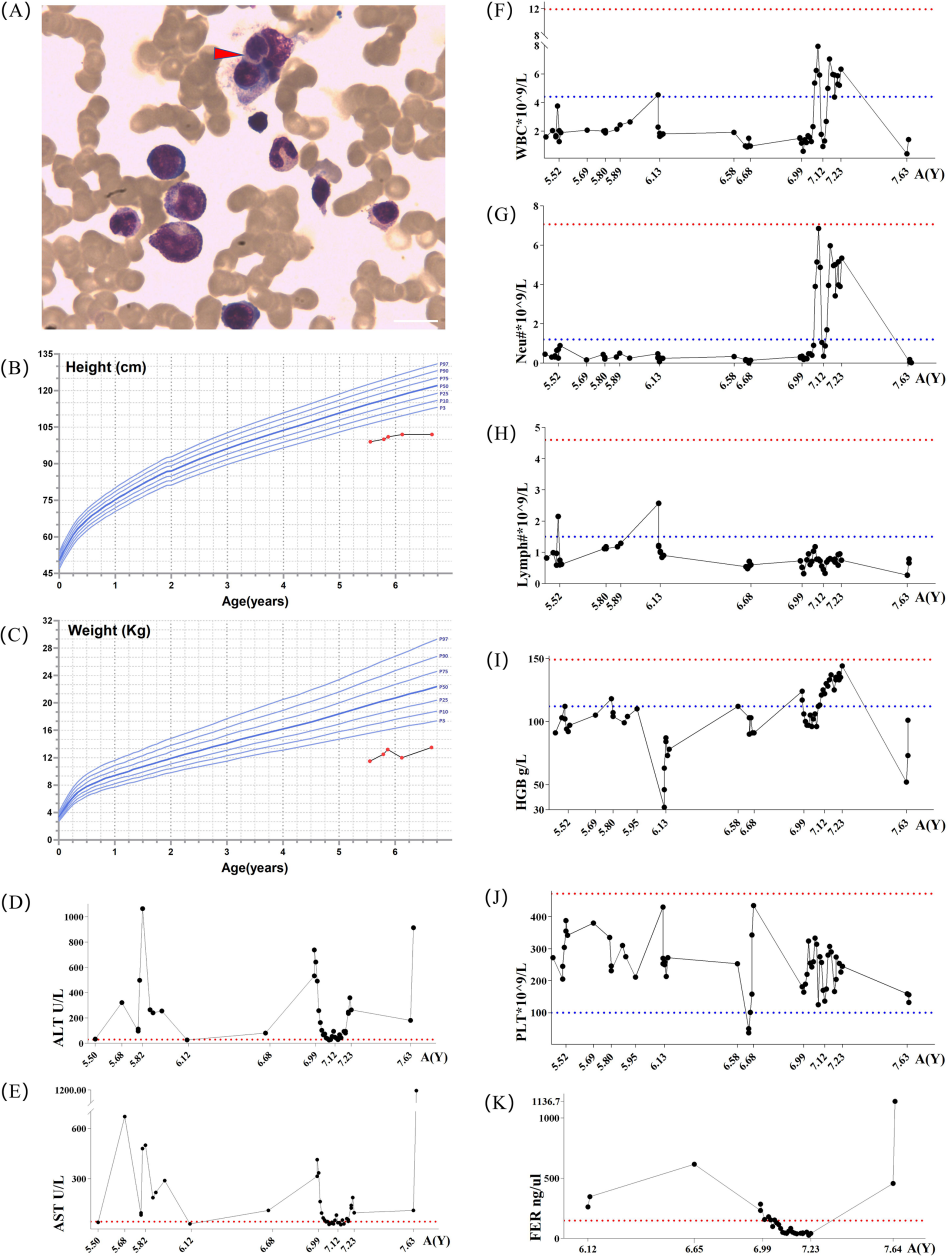
Cardinal clinical signs of the patient and dynamic trends of key parameters during the diagnostic and therapeutic course. **(A)** Bone marrow cytology; red triangular arrows indicate hemophagocytes. **(B, C)** Growth curves for height and weight. **(D–K)** Temporal variations in ALT, AST, WBC, Neu#, Lymph#, HGB, PLT, and FER. ALT, alanine aminotransferase; AST, aspartate aminotransferase; WBC, white blood cell count; Neu#, absolute neutrophil count; Lymph#, absolute lymphocyte count; HGB, hemoglobin; PLT, platelet count; FER, serum ferritin.

From 5 years and 8 months of age onward, she developed recurrent episodes of elevated serum aminotransferase levels, with or without fever. Between episodes, aminotransferase levels declined but did not return to the normal range. She also experienced two episodes of acute liver failure (ALF) and was clinically diagnosed with ILFS-2. At 6 years and 1 month, she developed severe anemia, with a hemoglobin (HGB) level of 32 g/L, and bone marrow cytology revealed markedly reduced erythroid lineage. At 6 years and 8 months, in the setting of persistent fever, she was diagnosed with HLH according to the HLH-2004 diagnostic criteria, based on fulfillment of five criteria: fever, splenomegaly, trilineage cytopenia, hyperferritinemia (serum ferritin, 615 μg/L), and hemophagocytosis in the bone marrow (**Figure 2A**). Abdominal ultrasound also revealed marked hepatomegaly.

At her first admission to our hospital, physical examination revealed short stature and malnutrition, with a height of 99 cm (<P3) and a weight of 11.5 kg (<P3). These findings were consistently observed at subsequent examinations (**Figures 2B, C**). Serial immunological assessments during multiple hospitalizations showed decreased NK cells and CD3^+^ T cells, a reduced CD4^+^/CD8^+^ ratio, and an increased proportion of B cells, whereas serum IgM, IgG, IgA, and IgE levels remained within the normal range (**Supplementary Tables 1**, **2**). After the diagnosis of HLH, serial serum cytokine profiling demonstrated recurrent elevations of multiple HLH-associated inflammatory mediators, including TNF-α, IFN-γ, IL-1β, IL-6, IL-12p70, and IL-10; IL-8 was also intermittently elevated (**Supplementary Table 3**). Psychomotor and intellectual development were normal. Cranial CT and ophthalmologic examinations were unremarkable. Repeated peripheral blood smears showed no Pelger–Huët anomaly. No facial dysmorphism or skeletal abnormalities were identified.

Before the diagnosis of HLH, the patient received anti-infective and supportive treatments, including hepatoprotective therapy, intravenous immunoglobulin (IVIG), and blood transfusions. Following the diagnosis of HLH, first-line therapy consisted of dexamethasone at 10 mg/m²/day plus ruxolitinib at 5 mg/m² per dose every 12 h, rather than a standard etoposide-based HLH protocol, because of the patient’s poor general condition. Ruxolitinib was administered for 8 weeks, whereas dexamethasone gradually tapered over a total course of approximately 16 weeks. Because the response remained inadequate, treatment was switched to emapalumab in combination with dexamethasone. During emapalumab therapy, emapalumab was administered at 1 mg/kg per dose twice weekly. During the early phase of emapalumab treatment, serum transaminase and ferritin levels decreased to the normal range, and hemoglobin, leukocyte, and neutrophil counts recovered to normal values, whereas lymphocyte counts remained below the lower limit of normal. In the later phase, leukocyte and neutrophil counts showed a transient decline due to infection. Four months later, emapalumab therapy was discontinued because of progressive primary HLH. After treatment withdrawal, the patient’s clinical condition deteriorated, with progressive increases in serum transaminase and ferritin levels and further decreases in hemoglobin, leukocyte, neutrophil, and lymphocyte counts (**Figures 2D–K**). Ultimately, the patient died of refractory HLH and ALF.

### Identification of NBAS mutations in the patient

**Figure 3:**
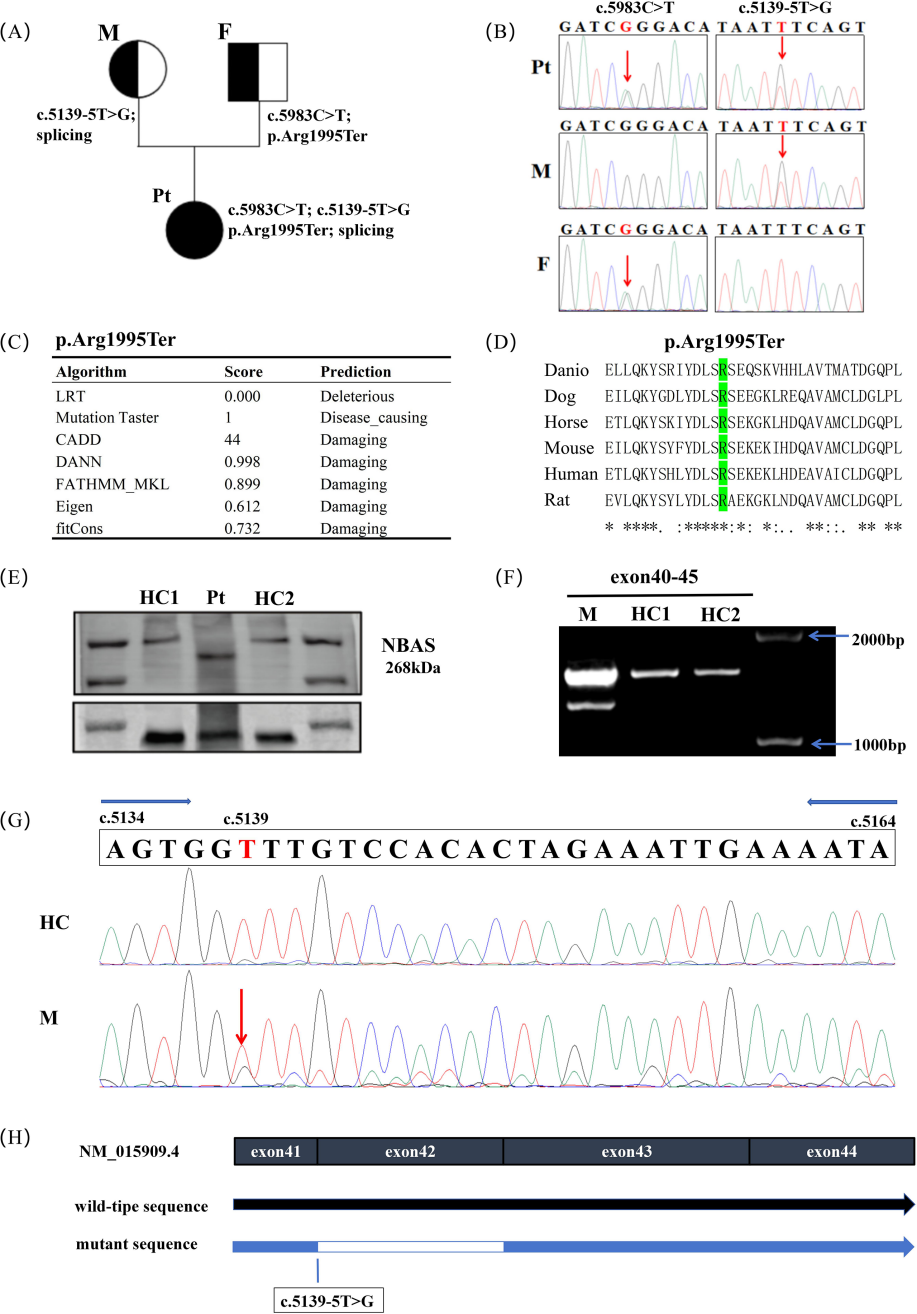
Genetic analysis of *NBAS* variants, NBAS protein expression in the patient, and aberrant splicing in the proband’s mother. **(A)** Family pedigree. **(B)** Sanger sequencing confirmed compound heterozygous variants in the *NBAS* gene: c.5139-5T>G (maternally inherited) and c.5983C>T (paternally inherited). **(C)** In silico pathogenicity prediction of p.Arg1995Ter. **(D)** Cross-species sequence alignment surrounding NBAS p.Arg1995 (highlighted in green). **(E)** NBAS protein expression in PBMCs from the patient and healthy controls; Pt, patient. **(F)** Agarose gel electrophoresis of PCR products spanning exons 40–45 of NBAS amplified from the mother’s cDNA and healthy controls; M, mother. **(G)** Compared with the controls, Sanger sequencing of the proband’s mother revealed overlapping peaks beginning at c.5139. **(H)** Alignment to the NBAS reference transcript (NM_015909.4) demonstrating that the aberrantly spliced transcript lacks exon 42.

To explaining the diverse clinical manifestations, WES was performed for the patient and her parents, which identified compound heterozygous variants in *NBAS* in the proband, c.5139-5T>G (splicing) and c.5983C>T (p.Arg1995Ter). The c.5139-5T>G variant was maternally inherited and c.5983C>T paternally inherited (**Figure 3A**), and both were confirmed by Sanger sequencing (**Figure 3B**). The c.5983C>T variant is novel and was classified as likely pathogenic based on the ACMG criteria, including PM2 (absent in population databases such as gnomAD, ExAC, and 1000 Genomes Project), PM3 (coexistence with a known pathogenic variant, c.5139-5T>G, in compound heterozygosity (recessive disease)), PM4(a nonsense variant leading to premature protein truncation), PP3 (LRT score=0, CADD score=44, DANN score=0.998, Mutation Taster score=1, FATHMM_MKL score=0.899, Eigen score=0.612, fitCons score =0.732, all of these predicted to be deleterious (**Figure 3C**)), and PP4 (the patient’s phenotype is highly consistent with NBAS-associated ILFS-2). Moreover, the arginine at position 1995 of the NBAS protein is highly conserved across species (**Figure 3D**).

### Functional studies of the identified NBAS variants

#### NBAS protein expression in PBMCs

To evaluate NBAS protein expression in the patient, we performed Western blot analysis using previously cryopreserved PBMCs. Western blotting of patient PBMCs lysates revealed only a single truncated NBAS protein band compared with healthy controls (**Figure 3E**), which corresponded to the NBAS protein with the c.5983C>T mutation, whose predicted molecular mass is approximately 220 kDa.

#### Splicing effect of the c.5139-5T>G variant

To assess the pathogenicity of the maternally inherited c.5139-5T>G variant, PCR was performed using cDNA generated from the peripheral blood of the proband’s mother. Agarose gel electrophoresis showed that, compared with unrelated controls, an additional shorter amplification product was detected in the proband’s mother (**Figure 3F**). Furthermore, Sanger sequencing revealed overlapping peaks beginning at c.5139 (**Figure 3G**), consistent with the presence of two distinct transcripts. PCR products corresponding to each band were gel-purified and sequenced separately. Sequencing of the lower band demonstrated skipping of NBAS exon 42 which contains 251 base pairs (bp) (**Figure 3H**). These findings demonstrate that the c.5139-5T>G variant disrupts normal NBAS mRNA splicing and results in loss of function.

#### The c.5983C>T variant reduces of NBAS mRNA expression

**Figure 4:**
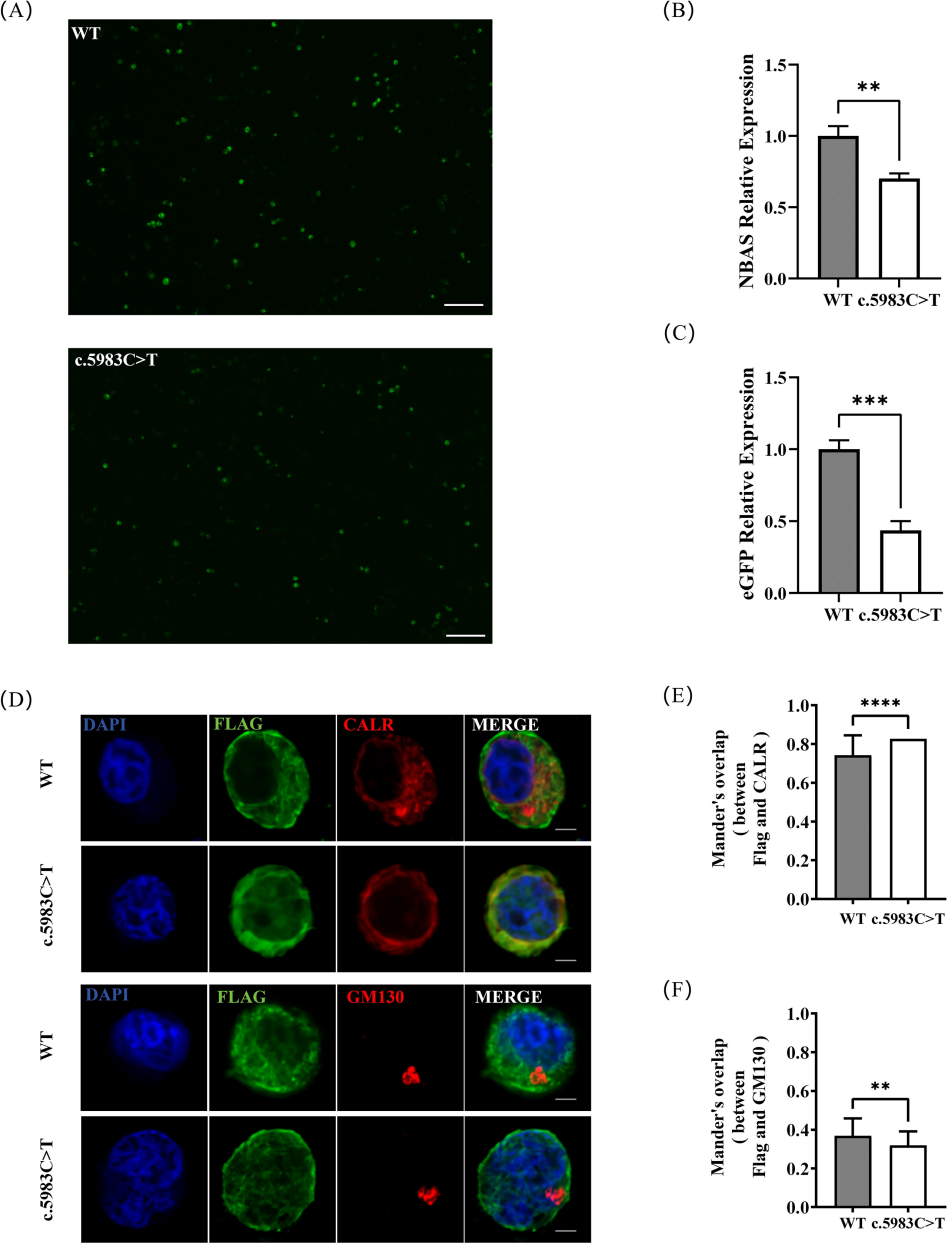
Functional studies of the p.Arg1995Ter variant in HEK293T cells. **(A)** Fluorescence micrographs of HEK293T cells 24 h after transfection with eGFP-tagged wild-type (WT) or mutant (MUT) NBAS constructs. **(B, C)** Relative NBAS mRNA levels in WT and MUT cells quantified using primers targeting NBAS and eGFP, normalized to 18S rRNA. **(D)** Representative images of transfected HEK293T cells stained for NBAS (anti-FLAG), endoplasmic reticulum (anti-calreticulin [CALR]), and Golgi apparatus (anti-GM130); nuclei are counterstained with DAPI (blue). Scale bars, 5 μm. **(E)** Manders’ overlap coefficients for colocalization of NBAS with the ER (WT: n = 59 cells; MUT: n = 71 cells). **(F)** Manders’ overlap coefficients for colocalization of NBAS with the Golgi (WT: n = 50 cells; MUT: n = 54 cells). A Student’s t-test was used for comparisons. Data are presented as mean ± SD, **P < 0.01, ***P < 0.001, ****P < 0.0001.

Given the presence of a truncated NBAS protein corresponding to the c.5983C>T variant in the patient, we generated a plasmid carrying the c.5983C>T mutation to further evaluate the effect of this variant on NBAS expression. We used a pcDNA3.1-eGFP expression vector containing WT or MUT NBAS constructs. After 24h of transfection, cells transfected with the MUT construct showed markedly reduced eGFP expression compared with those transfected with the WT construct (**Figure 4A**). Furthermore, NBAS mRNA expression was evaluated by RT-qPCR using primers targeting eGFP and NBAS. Both RT-qPCR analyzes demonstrated that NBAS mRNA expression in the MUT group was significantly reduced compared with the WT group (**Figures 4B, C**).

#### The c.5983C>T variant impairs Golgi-to-ER retrograde trafficking

Previous studies have shown that NBAS is involved in retrograde transport from the Golgi apparatus to the ER. To investigate the effect of the mutant NBAS protein on this pathway, we used a 7.1-pCMV-3×Flag expression vector containing WT or MUT NBAS constructs. Forty-eight hours after transfection, immunofluorescence analysis was performed to assess colocalization of NBAS with ER and Golgi markers. The MUT NBAS protein showed significantly increased colocalization with the ER compared with WT NBAS, whereas its colocalization with the Golgi apparatus was decreased (**Figures 4D–F**). These findings suggest altered vesicular trafficking between the Golgi and the ER.

### Literature review

#### Genotype–phenotype comparison

Given the unusually severe and complex phenotype of our patient, particularly the occurrence of fatal HLH in the setting of multisystem NBAS-associated disease, we reviewed previously reported cases to better define both the overall genotype–phenotype context and the specific relationship between NBAS deficiency and HLH. We compiled genotypic and clinical data from 100 publications and 5 conference abstracts, covering 322 patients. One patient identified only through genetic screening was excluded because the phenotype was incomplete due to very early antipyretic intervention, and five additional patients were excluded because of missing genotype data. The remaining patients were classified into the β-propeller, Sec39, and C-terminal groups according to the method proposed by Staufner et al (13), whereas patients not belonging to any of these three categories were assigned to the “Others” group. Major organ manifestations associated with NBAS deficiency were then analyzed, including hepatic, growth, optic, skeletal, neurologic, immune, hematologic, muscular, cutaneous, and craniofacial features.

**Figure 5:**
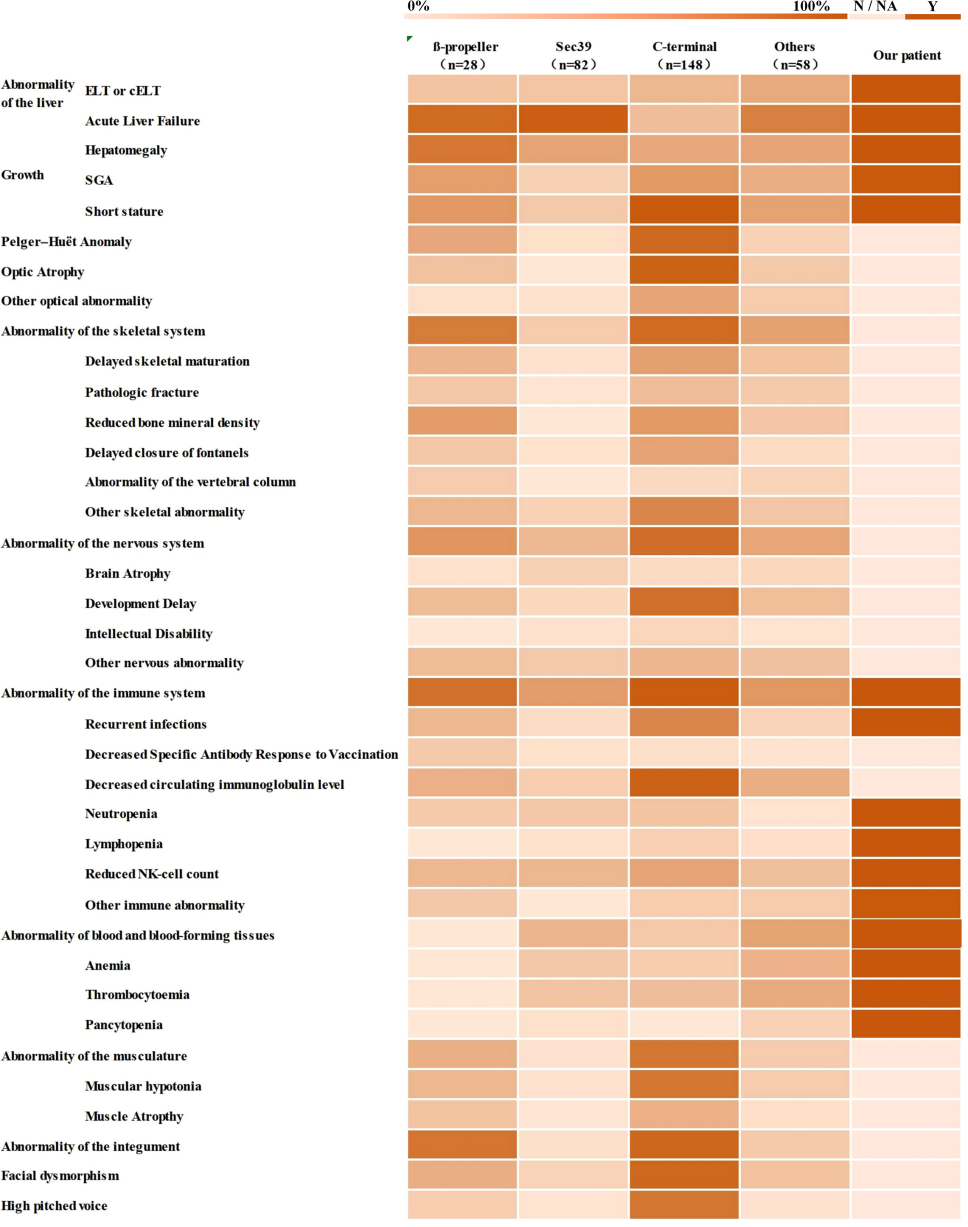
Genotype-phenotype features of 316 previously reported patients and the patient in this study. Patients were classified into the “β-propeller,” “Sec39,” and “C-terminal” groups according to the method proposed by Staufner et al.; patients who did not fall into any of these three categories were assigned to the “Others” group. On the left, bar color intensity indicates the percentage of patients displaying each feature. On the right, dark bars indicate features present in our patient, while light bars indicate features absent. ELT, elevated liver transaminases; cELT, continuously elevated liver transaminases; SGA, small for gestational age.

Distinct domain-associated clinical patterns were observed across the cohort. Patients with determining variants in the Sec39 domain (n = 82) showed a predominantly liver-dominant phenotype, with ALF representing the most characteristic manifestation (93.8%). Hepatomegaly, immune involvement, and hematologic abnormalities were also observed in a considerable proportion of patients, whereas involvement of other organ systems was comparatively infrequent. In contrast, patients with determining variants in the C-terminal domain (n = 148) exhibited a broad SOPH-like multisystem phenotype, characterized predominantly by short stature, Pelger–Huët anomaly, and optic atrophy. Skeletal, neurologic, immune, cutaneous, muscular, and craniofacial abnormalities were also highly prevalent in this group, whereas ALF was observed in only a minority of patients (28.6%). Patients with determining variants in the β-propeller domain (n = 28) showed a mixed phenotype, with frequent ALF (85.7%) and hepatomegaly (76.9%) accompanied by variable extrahepatic manifestations; notably, hematologic abnormalities were not observed in this subgroup. The “Others” group (n = 58) displayed a similarly hybrid phenotype, combining hepatic involvement with multisystem manifestations. In this subgroup, ALF and immune abnormalities were common, and hematologic abnormalities were also frequently encountered. Overall, these findings support distinct domain-associated genotype–phenotype correlations, with the Sec39 group showing a predominantly liver-dominant phenotype, the C-terminal group showing a SOPH-like multisystem phenotype, and the β-propeller and “Others” groups showing mixed phenotypes (**Figure 5**).

### Comparison with previously reported NBAS-deficient patients with HLH

To further highlight the relationship between NBAS deficiency and HLH, we compared our patient with the seven previously reported children with NBAS deficiency and HLH. Altogether, these eight patients harbored 15 distinct NBAS variants, including nine located in the C-terminal domain, three in the Sec39 domain, and three in the region between the β-propeller and Sec39 domains. According to the above classification, two patients were assigned to the C-terminal group and six to the “Others” group, suggesting that patients in these two categories may be more susceptible to developing HLH.

Clinically, previously reported cases were mainly characterized by HLH alone or HLH associated with liver dysfunction, with relatively limited extrahepatic involvement. Patient P87 (23) mainly presented with HLH, without additional organ involvement clearly attributable to NBAS deficiency. Patient P129 (24) showed HLH together with liver, skeletal, and neurologic involvement. Patient P259-P263 (22) were mainly characterized by HLH accompanied by liver dysfunction. By contrast, our patient exhibited a much broader and more severe phenotype, including pre- and postnatal growth retardation, immunodeficiency, ILFS-2, and progressive hematologic abnormalities, in addition to fatal HLH (**Table 1**). These findings suggest that, although uncommon, HLH may represent a severe inflammatory complication within the spectrum of NBAS-associated disease, particularly in patients with variants in the C-terminal or “Others” categories.

**Table 1:** Clinical manifestations of our patient and seven previously reported children with NBAS deficiency and HLH.

Clinical manifestations		Patients ID							
		Our pt	P87 (23)	P129 (24)	P259 (22)	P260 (22)	P261 (22)	P262 (22)	P263 (22)
Sex		F	M	F	F	F	M	F	M
Gene Mutation		c.5983C>T; c.5139-5T>G	c.2092T>C; c.2251G>A	c.1556T>A; c.1556T>A	c.1361C>G; c.6694C>G	c.3217C>T; c.5227A>G	c.4421C>T; c.4549A>G	c.4561G>C; c.6805G>A	c.3290C>T; c.4483T>C
Amino acid change		p.(R1995X); (splicing)	p.(Y698H); p.(D751N)	p.(V519E); p.(V519E)	p.(P454R); p.(Q2232E)	p.(R1073C); p.(M1743V)	p.(P1474L); p.(K1517E)	p.(E1521Q); p.(E2269K)	p.(T1097M); p. (Y1495H)
Abnormality of the liver	Hepatomegaly	Y	NA	Y	NA	NA	NA	NA	NA
	ALF	Y	N	Y	NA	NA	NA	NA	NA
	ELT/cELT	Y	N	N	Y	Y	Y	Y	Y
Growth	SGA	Y	N	Y	NA	NA	NA	NA	NA
	Short stature	Y	N	NA	NA	NA	NA	NA	NA
Optic Atrophy		N	NA	N	NA	NA	NA	NA	NA
Pelger–Huët Anomaly		N	NA	N	NA	NA	NA	NA	NA
Abnormality of the skeletal system		NA	NA	Four fractures without trauma	NA	NA	NA	NA	NA
Abnormality of the nervous system		N	N	Frontal atrophy; non-hemorrhagic fluid collection in the occipital area; Delayed milestones	NA	NA	NA	NA	NA
Abnormality of the immune system		Repeated infection; Leukopenia, neutropenia, and lymphocytopenia; Reduced NK cell count, CD3^+^ cell count and CD4^+^/CD8^+^ ratio; increased CD19^+^ percentage;	N	Low Ig	N	N	Autoinflammatory disease	N	N
Abnormality of the musculature		N	NA	NA	NA	NA	NA	NA	NA
Abnormality of the integument		N	NA	NA	NA	NA	NA	NA	NA
Facial dysmorphism		N	NA	N	NA	NA	NA	NA	NA
HLH diagnostic criteria	Fever	Y	Y	Y	Y	Y	Y	Y	Y
	Splenomegaly	Y	NA	Y	Y	N	N	Y	N
	Cytopenia	Pancytopenia	Erythropenia Thrombocytopenia	Erythropenia	Pancytopenia	Pancytopenia	Leukopenia Erythropenia;	Erythropenia Thrombocytopenia	Pancytopenia
	Hemophagocytosis in bone marrow	Y	NA	NA	Y	Y	Y	Y	Y
	Hypertriglyceridemia	Y	Y	Y	Y	Y	N	N	N
	Hypofibrinogenemia	N	Y	NA	N	N	N	N	Y
	Low NK cell activity	Y	N	NA	Y	Y	Y	Y	Y
	Hyperferritinemia (ug/L)	1136.7	2880	18 850	1390.5	42400	54886	5965	4440
	Elevated soluble CD25 (pg/ml)	NA	NA	NA	Y	Y	Y	Y	Y
Other HLH abnormalities	CNS involvement	N	Y	N	N	N	N	N	N
	EBV infection	N	N	NA	Y	Y	N	Y	Y
	Cytokine storm	Y	N	NA	NA	NA	NA	NA	NA
Outcome		Died	Alive	Alive	LTF	Alive	Alive	LTF	Alive

Patients ID, identifiers of all 322 previously reported patients included in our review; Our pt, Our patient; P, patient; F, female; M, male; Y, yes; N, no; NA, not available; ALF, acute liver failure; ELT, elevated liver transaminases; cELT, continuously elevated liver transaminases; SGA, small for gestational age; LTF, Lost to follow-up.

## Discussion

NBAS were firstly reported as a cause of SOPH syndrome in Yakuts. To date, 322 patients were published and biallelic variants in NBAS gene result in marked clinical heterogeneity, giving rise to a broad spectrum of growth, skeletal, ocular, immunologic, and hepatic manifestations. However, most patients exhibit only a subset of these features. Here, we describe a patient with novel pathogenic NBAS mutations who exhibited a clinical phenotype distinct from those previously described. The patient was born small for gestational age, and presented with recurrent upper respiratory tract infections and tonsillitis at 1 year of age, diagnosed with growth faltering at 4 years of age, developed leukopenia and acute liver failure at 5 years, progressed to severe pancytopenia and HLH at 6 years, and ultimately died of refractory HLH at 7 years and 8 months of age.

NBAS-associated disease exhibits substantial phenotypic heterogeneity, and published cohorts and case series most consistently describe ILFS-2, SOPH syndrome, and variable immune dysfunction (5, 13, 61). Immune-related hematologic abnormalities—most notably neutropenia and broader immune cytopenias in the setting of immunodeficiency—have also been reported; however, persistent trilineage cytopenia or overt pancytopenia is not typically presented as a defining feature of NBAS-related disorders in genotype–phenotype studies and immunophenotyping reports (13, 53). Rather, severe pancytopenia is a well-recognized manifestation of hyperinflammatory states such as HLH, and is frequently encountered in the context of severe infection and organ dysfunction during cytokine-driven immune activation (22, 75).

In line with this framework, the temporal association in our patient—progression from recurrent leukopenia to pancytopenia emerging alongside HLH—supports the interpretation that bone marrow failure reflected escalating systemic immune dysregulation rather than an intrinsic, stable hematologic phenotype attributable to NBAS deficiency. Although HLH is not considered a hallmark feature of NBAS-associated disease, recent evidence indicates that biallelic NBAS variants can be identified in pediatric HLH cohorts and have also been documented in individual cases, suggesting that HLH represents an uncommon but clinically critical complication in a subset of patients (21–24).

Once HLH develops, available evidence and consensus guidance emphasize its potential for rapid clinical deterioration with progressive cytopenias, hepatic involvement, and high mortality risk (22, 75). Accordingly, the fatal outcome in our case underscores refractory HLH as a major proximate driver of mortality when this complication arises. Treating HLH in the context of NBAS deficiency may be particularly challenging. First, these patients often present with multisystem involvement, especially severe liver dysfunction, recurrent infections, and progressive cytopenias, all of which may limit tolerance to standard etoposide-based HLH protocols. In our patient, poor general condition and marked hepatic injury precluded the initial use of a conventional etoposide-based regimen, and an individualized anti-inflammatory strategy was selected instead. Second, although targeted agents such as ruxolitinib and emapalumab may offer a less myelotoxic alternative for controlling hyperinflammation, their use in profoundly immunocompromised patients may still be complicated by recurrent or opportunistic infections. In our case, transient hematologic improvement and normalization of ferritin and transaminase levels were achieved during the early phase of emapalumab therapy, but treatment was later interrupted because of repeated infections, followed by rapid clinical deterioration. These observations highlight the therapeutic dilemma in NBAS-associated HLH: effective control of hyperinflammation is urgently required, yet treatment intensification may be constrained by underlying organ dysfunction, baseline cytopenias, and infection susceptibility. Our findings underscore the importance of early recognition, individualized therapeutic strategies, and aggressive management when HLH complicates NBAS-associated disease.

*NBAS* encodes a highly conserved protein with an established role in Golgi-to-ER retrograde trafficking (2, 3), and loss-of-function variants in NBAS are associated with diverse clinical phenotypes (12, 19, 31, 48, 64). In this study, we identified compound heterozygous *NBAS* variants consisting of splice-site mutation c.5139-5T>G and a novel truncating variant c.5983C>T, both of which impair NBAS function. The c.5139-5T>G variant has previously been described in a Chinese boy diagnosed with autoimmune thyroiditis and autoimmune thrombocytopenia (76). However, no functional validation of pathogenicity was performed. Based on our experimental results, the c.5139-5T>G variant causes aberrant splicing. The splicing-mutant NBAS transcript is predicted to produce a protein of ~251 kDa based on the sequencing results. However, the predicted band was absent in Western blotting of the patient’s PBMCs, indicating that the mutant NBAS transcript likely undergoes NMD. The pathogenicity of the newly identified c.5983C>T variant is supported by its absence from population databases, the high evolutionary conservation of the affected residue, and experimental evidence demonstrating reduced NBAS mRNA abundance and expression of a truncated protein. Functional analyzes further showed altered subcellular localization of the mutant protein, consistent with disrupted vesicular trafficking rather than complete loss of protein expression.

Notably, beyond its direct role in Golgi-to-ER retrograde transport, NBAS deficiency may also induce ER-stress. Haack et al. reported that fibroblasts from individuals with biallelic NBAS variants showed markedly reduced levels of NBAS and its interaction partner p31, indicating dysfunction of the ER–Golgi retrograde transport complex, together with upregulation of ER stress response genes (5). Recent studies of Golgi-trapped CDC42 variants have shown that abnormal intracellular localization can disrupt ER–Golgi trafficking, induce ER stress, and contribute to autoinflammatory phenotypes (77, 78). By analogy, defective NBAS may also contribute to severe inflammatory phenotypes through a mechanism linking ER–Golgi trafficking defects, ER stress, and inflammatory amplification. In our proband with HLH, persistent cytopenias, and severe liver injury, such a pathway may represent a plausible explanation for the development of hyperinflammation, although further studies are needed to confirm this hypothesis.

In conclusion, we identify a novel pathogenic NBAS variant that further supports the essential role of NBAS in cellular homeostasis. Clinically, our findings indicate that although pancytopenia and HLH are not defining features of NBAS-associated disease, their emergence signals severe immune dysregulation and is associated with poor prognosis. Early recognition of these complications may be critical for timely intervention and outcome stratification in affected patients.

## Data Availability

The original contributions presented in the study are included in the article and **Supplementary Material**. The raw individual-level genetic data are not publicly available due to privacy and ethical restrictions. Further inquiries can be directed to the corresponding author.
