# Supplementary material for: A novel compound heterozygous NBAS variant with HLH and multisystem involvement: expanding the clinical spectrum and literature review
Source: Front Immunol. 2026 Jun 1;17:1829362. doi: 10.3389/fimmu.2026.1829362 (PMC13265293; doi:10.3389/fimmu.2026.1829362)
Supplement: Supplementary file 1 [file Table1.docx]

**Supplementary Table 1** Summary of the patient’s serial TBNK results

| AGE（Year） | CD3^+^%  (55-78) | CD3^+^CD8^+^%  (19-34) | CD3^+^CD4^+^%  (27-53) | CD3^+^CD4^+^CD8^+^% | CD3^+^CD4-CD8-% | NK%  (4-26) | CD19^+^%  (10-31) | CD4/CD8  (0.98-1.94) | CD3^+^count  Cells/ul  (700-4200) | CD3^+^CD8^+^count  Cells/ul  (300-1800) | CD3^+^CD4^+^count  Cells/ul  (300-2000) | CD3^+^CD4^+^CD8^+^count  Cells/ul | NKcount  Cells/ul  (90-900) | CD19^+^count  Cells/ul  (20-1600) | CD45^+^Count  Cells/ul |
| --- | --- | --- | --- | --- | --- | --- | --- | --- | --- | --- | --- | --- | --- | --- | --- |
| 5.50 | 52.39 | 29.75 | 20.2 | 0.46 | 2.9 | 11.78 | 35.79 ↑ | 0.68 ↓ | 282.17 ↓ | 160.21 | 128.78 | 2.49 | 63.42 ↓ | 192.74 | 538.57 |
| 5.87 | 57.54 | 32.54 | 22.35 | 0.34 | 2.99 | 8.29 | 34.14 ↑ | 0.69 ↓ | 569.68 ↓ | 322.19 | 221.27 | 3.4 | 82.1 ↓ | 338.01 | 989.9 |
| 6.65 | 53.41 | 24.68 | 17.3 | 2.08 | 13.51 | 24.63 | 21.96 | 0.7 ↓ | 81.71 ↓ | 37.75 | 26.46 | 3.18 | 37.68 ↓ | 33.59 | 152.97 |

**Supplementary Table 2** Summary of the patient’s serial measurement of immunoglobulin levels

| AGE（Year） | IgG（g/L）  (5.28-21.9) | IgA（g/L）  (0.43-2.53) | IgM（g/L）  (0.48-2.26) | IgE (IU/ml)  (0-165) | C3（g/L）  (0.7-2.06) | C4（g/L）  (0.11-0.61) |
| --- | --- | --- | --- | --- | --- | --- |
| 5.50 | 12.4 | 0.884 | 1.04 | 57 | 1.42 | 0.3 |
| 5.87 | 18.1 | 1.13 | 0.707 | 21.8 | 1.25 | 0.27 |
| 6.13 | 30.4 | 1.38 | 0.878 | 37.5 | 1.1 | 0.22 |

| AGE（Year） | IL-2(g/ml)  (0-9.8) | IL-4(g/ml)  (0-3.0) | IL-6(g/ml)  (0-16.6) | IL-10(g/ml)  (0-4.9) | TNF-α(g/ml)  (0-5.2) | INF-γ(g/ml)  (0-17.3) | IL-17A(g/ml)  (0-14.8) | IL-1β(g/ml)  (0-12.4) | IL-5(g/ml)  (0-3.1) | IL-12p70(g/ml)  (0-3.4) | TNF-α(g/ml)  (0-8.5) | IL-8(g/ml)  (0-20.6) | CCL5(g/ml)  (0-4215.7) |
| --- | --- | --- | --- | --- | --- | --- | --- | --- | --- | --- | --- | --- | --- |
| 5.50 | 0 | 0.3 | 6.29 | 14.53 ↑ | 0.13 | 0 | 0 | 0.19 | 1.41 | 0 | 12.46 | 20.94 |  |
| 6.65 | 1.22 | 59.68 ↑ | 1134.35↑ | 29.97 ↑ | 19.26 ↑ | 45.49 ↑ | 3.69 | 27.75 ↑ | 5.81 ↑ | 1.71 | 524.22 ↑ | 262.6 ↑ |  |
| 7 | 1.53 | 3.35 ↑ | 1.28 | 10.98 ↑ | 12.91 ↑ | 0.74 | 1.89 | 11.03 | 1.33 | 0.72 | 26.6 ↑ | 17.34 |  |
| 7.02 | 0.6 | 2.25 | 1.31 | 5.19 ↑ | 11.64 ↑ | 0.61 | 0 | 13.26 ↑ | 1.37 | 0.38 | 28.67 ↑ | 9.8 |  |
| 7.04 | 1.53 | 4.97 ↑ | 12.37 | 23.45 ↑ | 7.77 ↑ | 1.78 | 12.58 | 8.83 | 1.31 | 3.99 ↑ | 21.85 ↑ | 18.08 |  |
| 7.05 | 0.17 | 1.05 | 2.27 | 5.94 ↑ | 3.92 | 0.53 | 1.7 | 3.86 | 0.39 | 0.04 | 11.28 ↑ | 12.61 |  |
| 7.06 | 0.12 | 1.07 | 1.55 | 3.08 | 3.98 | 0 | 0 | 3.56 | 0.26 | 0 | 16.14 ↑ | 8.12 |  |
| 7.07 | 0.16 | 1.01 | 1.33 | 3.26 | 2.03 | 0 | 4.14 | 2.29 | 0.08 | 0 | 12.35 ↑ | 5.77 |  |
| 7.08 | 0.12 | 0.61 | 0.65 | 2.02 | 2.70 | 0.09 | 0 | 1.57 | 0.27 | 0 | 11.46 ↑ | 3.95 |  |
| 7.09 | 0.15 | 0.35 | 0.96 | 2.8 | 1.25 | 0.05 | 6.18 | 1.59 | 0.22 | 0 | 8.05 | 3.88 |  |
| 7.10 | 0 | 0 | 0.36 | 2.96 | 0.97 | 0.06 | 0 | 1.43 | 0.04 | 0 | 7.82 | 2.56 |  |
| 7.14 | 0 | 2.36 | 1.13 | 4.67 | 4.16 | 0 | 12.49 | 5.83 | 0.52 | 1.44 | 30 ↑ | 10.82 | 10513.35 ↑ |
| 7.63 | 1.83 | 5.62 ↑ | 15.91 | 22.02 ↑ | 3.1 | 1.26 | 5.79 | 5.6 | 0.55 | 3.64 ↑ | 9.7 ↑ | 62.91 ↑ |  |

**Supplementary Table 3** Serial cytokine testing results in the patient
